# Comparisons of Natural and Cultivated Populations of *Corydalis yanhusuo* Indicate Divergent Patterns of Genetic and Epigenetic Variation

**DOI:** 10.3389/fpls.2020.00985

**Published:** 2020-07-03

**Authors:** Chen Chen, Zhi Zheng, Yiqiong Bao, Hanchao Zhang, Christina L. Richards, Jinghui Li, Yahua Chen, Yunpeng Zhao, Zhenguo Shen, Chengxin Fu

**Affiliations:** ^1^ College of Life Sciences, Nanjing Agricultural University, Nanjing, China; ^2^ Department of Integrative Biology, University of South Florida, Tampa, FL, United States; ^3^ Plant Evolutionary Ecology Group, University of Tübingen, Tübingen, Germany; ^4^ Laboratory of Systematic and Evolutionary Botany and Biodiversity, and College of Life Sciences, Zhejiang University, Hangzhou, China

**Keywords:** DNA methylation, epigenetic variation, sexual and asexual reproduction, *Corydalis yanhusuo*, environmental response

## Abstract

Epigenetic variation may contribute to traits that are important in domestication, but how patterns of genetic and epigenetic variation differ between cultivated and wild plants remains poorly understood. In particular, we know little about how selection may shape epigenetic variation in natural and cultivated populations. In this study, we investigated 11 natural populations and 6 major cultivated populations using amplified fragment length polymorphism (AFLP) and methylation-sensitive AFLP (MS-AFLP or MSAP) markers to identify patterns of genetic and epigenetic diversity among *Corydalis yanhusuo* populations. We further explored correlations among genetic, epigenetic, alkaloidal, and climatic factors in natural and cultivated *C. yanhusuo*. We found support for a single origin for all cultivated populations, from a natural population which was differentiated from the other natural populations. The magnitude of *F*_ST_ based on AFLP was significantly correlated with that for MSAP in pairwise comparisons in both natural and cultivated populations, suggesting a relationship between genetic and epigenetic variation in *C. yanhusuo*. This relationship was further supported by dbRDA (distance-based redundancy analyses) where some of the epigenetic variation could be explained by genetic variation in natural and cultivated populations. Genetic variation was slightly higher in natural than cultivated populations, and exceeded epigenetic variation in both types of populations. However, epigenetic differentiation exceeded that of genetic differentiation among cultivated populations, while the reverse was observed among natural populations. The differences between wild and cultivated plants may be partly due to processes inherent to cultivation and in particular the differences in mode of reproduction. The importance of epigenetic compared to genetic modifications is thought to vary depending on reproductive strategies, and *C. yanhusuo* usually reproduces sexually in natural environments, while the cultivated *C. yanhusuo* are propagated clonally. In addition, alkaloid content of *C. yanhusuo* varied across cultivated populations, and alkaloid content was significantly correlated to climatic variation, but also to genetic (6.89%) and even more so to epigenetic (14.09%) variation in cultivated populations. Our study demonstrates that epigenetic variation could be important in cultivation of *C. yanhusuo* and serve as a source of variation for response to environmental conditions.

## Introduction

Cultivated plants possess major morphological and physiological changes compared to their wild progenitors (Darwin, 1868). Moreover, many quantitative traits in cultivated plants can become differentiated across the cultivated range due to selection in new environments and in response to different human preferences (Purugganan and Fuller, 2009; Fuller et al., 2011; Meyer and Purugganan, 2013). The wide range of possible phenotypes results from continuous rounds of artificial selection despite the fact that cultivated plants are presumed to have genetic bottlenecks inherent to strong artificial selection and genetic drift (Hyten et al., 2006; Berger et al., 2012; Meyer and Purugganan, 2013; Verde et al., 2013). While most studies of the inheritance of phenotypic differences in cultivated species have focused on DNA sequence differences (Caicedo et al., 2007; Hufford et al., 2012; Meyer and Purugganan, 2013; Lin et al., 2014; Zhou et al., 2015), recent work suggests that some of this increased phenotypic diversity could be epigenetically based (Hauben et al., 2009; Ji et al., 2015; Gallusci et al., 2017; Shen et al., 2018).

DNA methylation is one epigenetic mechanism that could contribute to organismal response to environmental conditions since DNA methylation modifications can affect transcription, and lead to phenotypic variation (Nicotra et al., 2010; Wibowo et al., 2016; Richards et al., 2017). Studies in both plant and animal species with low levels of genetic diversity have found that variation in DNA methylation was correlated to habitat type, exposure to stress, and shifts in species range suggesting that environmentally induced epigenetic changes may contribute to the ability to cope with environmental variation (Richards et al., 2012; Verhoeven and Preite, 2014: Liebl et al., 2015; Xie et al., 2015). Further, several studies have found that epigenetic variation is not entirely explained by genetic variation, and that the amount of epigenetic variation often exceeds that of sequence variation among natural populations (e.g., *Viola cazorlensis*, Herrera and Bazaga, 2010; *Laguncularia racemose,* Lira-Medeiros et al., 2010; *Fallopia* spp., Richards et al., 2012; *Viola elatior*, Schulz et al., 2014). However, deciphering the relationship between genetic and epigenetic variation remains a significant challenge (Schulz et al., 2014; Alonso et al., 2016; Foust et al., 2016; Gugger et al., 2016; Herrera et al., 2017; Richards et al., 2017), and some studies have found that DNA methylation polymorphisms are explained by underlying genetic differences (Becker et al., 2011; Li et al., 2014; Dubin et al., 2015), and therefore do not provide any additional sources of phenotypic variation. Although epigenetic variation among wild plant populations has been well studied, the changes in epigenetic variation involved in cultivation of plants remain poorly understood (Piperno, 2017; Lu et al., 2020). In addition to studies comparing domesticated species to their wild progenitors (Li et al., 2012; Sauvage et al., 2017), studies of crop plants with both natural and cultivated populations will shed light on the importance of epigenetic and genetic variation in domestication.

Vegetative propagation has been a customary technique in modern cultivation, including for economically important crops like manioc, potato, sugarcane, sweet potato, taro, and yams (Denham et al., 2020). Many of the economically important crops that are vegetatively propagated, are derived from wild type progenitors that largely reproduce sexually (Denham et al., 2020). While recent evidence is equivocal about the loss of genetic diversity through domestication for outcrossing grain crops (Meyer and Purugganan, 2013; Allaby et al., 2019), genetic diversity in vegetatively propagated plants can be much lower and many vegetatively propagated plants lose the capacity for sexual reproduction (Denham et al., 2020). As for many clonal species, phenotypic plasticity may also be important for clonally propagated crops (Denham et al., 2020), and such plasticity may be mediated by epigenetic effects (Herman et al., 2014; Herman and Sultan, 2016; Richards et al., 2017; Banta and Richards, 2018). In fact, previous studies on a variety of species have revealed that the dynamics of plant responses that are mediated by DNA methylation may differ between sexual and asexual plants (Verhoeven and Preite, 2014). This could be partly due to the fact that much of DNA methylation is reset during sexual reproduction of plants (Jullien and Berger, 2010; Gehring, 2019), which may restrict the transmission to the next generation of epigenetic variation caused by environmental induction (Feng and Jacobsen, 2011; Heard and Martienssen, 2014; Tricker, 2015; Wibowo et al., 2018). However, such reprogramming is bypassed in plants that reproduce asexually, suggesting that epigenetic changes induced by environmental conditions could be maintained in asexual plants over generations (Richards et al., 2012; Verhoeven and Preite, 2014; Dodd and Douhovnikoff, 2016; González et al., 2018). This maintenance of epigenetic variation through asexual reproduction may be important in the process of adaptation, contributing to the short-term evolution of asexual plants (Verhoeven and Preite, 2014; Dodd and Douhovnikoff, 2016). Thus, the role of DNA methylation in adaptation may differ between wild and clonally propagated crops, but the comparison of genetic and epigenetic variation among sexual and asexual crops has not yet been fully investigated.

*Corydalis yanhusuo* W.T. Wang ex Z.Y. Su et C.Y. Wu is a diploid (2*n* = 32), perennial herb endemic to China (Xu and Wei, 1994; Qiu et al., 2009). The well-known traditional Chinese medicine, *Rhizoma corydalis* is made from the dried rhizomes of *C. yanhusuo* which contains several important alkaloids that promote blood activation and pain relief (Pharmacopoeia Committee of P. R. China, 2010). The concentrations of the alkaloids tetrahydropalmatine, protopine, palmatinem, and berberine are the most likely phenotypes under selection for these medicinal purposes. At present, *C. yanhusuo* is planted widely across China, with several major *C. yanhusuo*-producing regions in Zhejiang, Shanxi, and Jiangsu provinces. The cultivation history of *C. yanhusuo* can be traced back to the Ming Dynasty when it was originally planted in Zhejiang Province and then gradually introduced to other regions of China (Qiu et al., 2009). In the production of cultivated *C. yanhusuo*, the tubers are usually planted in October, propagated through asexual reproduction, and harvested in May before flowering or fruit set. On the other hand, in natural environments *C. yanhusuo* usually reproduces sexually (Qiu et al., 2009). Due to human overexploitation and environmental degradation, natural *C. yanhusuo* populations are decreasing, and its range is shrinking. At present, the natural distribution of *C. yanhusuo* is limited to hilly areas in the middle and lower reaches of the Yangtze River. In this study, we investigated natural and cultivated populations of *C. yanhusuo* to examine how genetic and epigenetic variation is correlated to climatic differences in the two types of populations. Using epigenetic, genetic, environmental, and phenotypic (alkaloids) data we: (1) examined the differences between natural and cultivated *C. yanhusuo* in genetic and epigenetic variation and climatic conditions; and (2) explored the association between genetic, epigenetic, and climatic variation and alkaloid content in cultivated *C. yanhusuo*. With these data, we tested the hypothesis that genetic differentiation is lower and epigenetic differentiation is higher in cultivated than natural populations. We also tested the hypothesis that epigenetic variation is predicted partly by climatic conditions and highly correlated to alkaloid production in cultivated populations supporting the idea that epigenetic variation is an important source of phenotypic variation in cultivated plants.

## Materials and Methods

### Plant Materials and DNA Isolation

**Figure 1:**
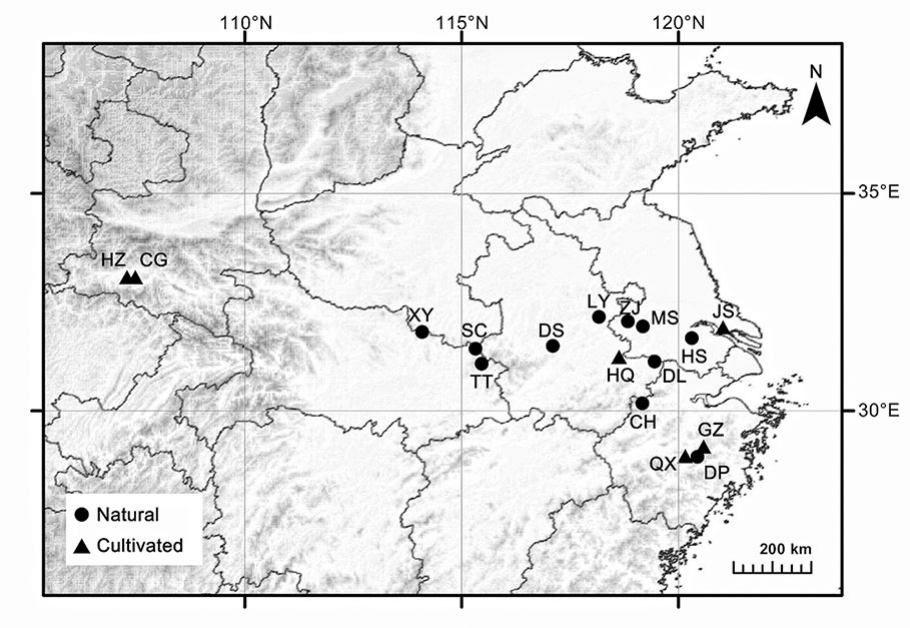
Sampling locations of *Corydalis yanhusuo* populations. The circle represents natural populations and the triangle refers to cultivated populations.

According to the Flora of China (http://www.efloras.org/index.aspx), *C. yanhusuo* has two synonyms: *C. turtschaninovii* Besser f. *yanhusuo* Y. H. Chou & C. C. Hsu and *C. tertici* (Nakai) Nakai f. *yanhusuo* (Y. H. Chou & C. C. Hsu) Y. C. Zhu. In this study, we collected leaves of approximately 15 to 20 individuals of wild *C. yanhusuo* from the 11 known natural populations (**Figure 1**; **Table 1**). The distances between neighboring plants exceeded 15 m. In addition, we obtained leaves and rhizomes from 14 to 20 individuals of *C. yanhusuo* from 6 cultivated populations representing the major *C. yanhusuo*-producing regions in China (CG, GZ, HQ, HZ, JS, and QX; **Figure 1**, **Table 1**). Leaf tissues were ground in liquid nitrogen, and genomic DNA was extracted using DNA Plantzol Reagent (Invitrogen, Carlsbad, CA, USA) according to the manufacturer’s protocol. The rhizomes from cultivated populations were dried at 60° C to a constant weight and prepared for alkaloid analysis.

**Table 1:** The epigenetic and genetic diversity index across the 17 populations of *Corydalis yanhusuo*.

Population	Longitude/Latitude	Locality	Number	*H*’_AFLP_ (SE)	H’MSAP(SE)
**Cultivated**					
CG	107°16’12”E/33°4’48”N	Hujiawan, Chenggu county, Shanxi province	18	0.3232 (0.0118)	0.1537 (0.0072)
GZ	120°28’48”E/29°7’48”N	Guzhu, Panan county, Zhejiang province	20	0.2710 (0.0120)	0.1420 (0.0071)
HQ	118°37’12”E/31°15’11”N	Huaqiao, Wuhu county, Anhui province	20	0.3273 (0.0123)	0.1447 (0.0072)
HZ	107°28’12”E/33°4’48”N	Hanzhong city, Shanxi province	14	0.3160 (0.0121)	0.1206 (0.0066)
JS	121°1’12”E/31°55’48”N	Nantong city, Jiangsu province	20	0.3058 (0.0117)	0.1650 (0.0075)
QX	120°19’48”E/29°1’48”N	Qianxiang, Dongyang city, Zhejiang province	20	0.2789 (0.0121)	0.1559 (0.0074)
Overall			112	0.3037 (0.0049)	0.1470 (0.0029)
**Natural**					
CH	119°9’36”E/30°10’12”N	Changhua, Linan city, Zhejiang province	15	0.1799 (0.0095)	0.0920 (0.0063)
DL	119°26’24”E/31°8’24”N	Dalong, Yixing City, Jiangsu province	18	0.4338 (0.0110)	0.0795 (0.0057)
DP	120°25’48”E/29°3’12”N	Dapan Mountain, Panan county, Zhejiang province	20	0.2605 (0.0112)	0.1514 (0.0076)
DS	117°14’23”E/31°30’15”N	Dashu mountain, Hefei city, Anhui province	17	0.4507 (0.0108)	0.0747 (0.0055)
HS	120°16’48”E/31°40’12”N	Huishan county, Wuxi city, Jiangsu province	20	0.3124 (0.0120)	0.1419 (0.0071)
XY	114°5’24”E/31°48’36”N	Jigong mountain, Xinyang city, Henan province	16	0.3470 (0.0115)	0.0893 (0.0061)
SC	115°19’12”E/31°25’48”N	Jingangtai, Shangcheng county, Henan province	20	0.4046 (0.0118)	0.1059 (0.0065)
LY	118°9’36”E/32°9’36”N	Langya mountain, Chuzhou city, Anhui province	18	0.4648 (0.0112)	0.1351 (0.0071)
MS	119°10’12”E/31°57’11”N	Mao mountain, Jurong city, Jiangsu province	20	0.2986 (0.0102)	0.1061 (0.0064)
TT	115°27’06”E/31°5’24”N	Tiantangzhai, Jinzhai county, Anhui province	17	0.3774 (0.0124)	0.0849 (0.0058)
ZJ	118°49’48”E/32°3’36”N	Zijin mountain, Nanjing city, Jiangsu province	20	0.3167 (0.0116)	0.1359 (0.0071)
Overall			201	0.3497 (0.0036)	0.1088 (0.0020)

H′_AFLP_, Shannon’s diversity for AFLP data; H′_MSAP_, Shannon’s diversity for MSAP data. SE, standard errors.

### Molecular Analysis

We screened 201 individuals of *C. yanhusuo* from 11 natural populations and 112 individuals from 6 cultivated populations for analysis of genetic and epigenetic variation. We used the amplified fragment length polymorphism (AFLP) technique of Vos et al. (1995) to analyze genetic variation. Genomic DNA was digested with *EcoR*I/MseI, followed by pre- and selective amplification. Eight fluorescently labeled primer pairs were used for selective amplification (**Table S2**), and the products were resolved by Genewiz, Inc. (Suzhou, China) on an ABI3730 instrument. Fragments of 69 to 450 bp were identified using GENEMARKER ver. 2.2.0 (SoftGenetics, State College, PA, USA), and transformed into a binary character matrix, with “1” indicating presence and “0” indicating absence.

We used the methylation-sensitive AFLP (MS-AFLP or MSAP) method of Reyna-López et al. (1997) with slight modification (Shi et al., 2017) to analyze epigenetic variation. MSAP is similar to AFLP except for double digestion with *EcoR*I/*Msp*I or *EcoR*I/*Hpa*II in place of *EcoR*I/*Mse*I as the frequent cutter. *Msp*I and *Hpa*II are isoschizomers, but have different sensitivities to methylation of cytosine within the tetranucleotide recognition sequence 5′-CCGG-3′. Cleavage by *Msp*I is blocked if the outer cytosines are hemi- or fully methylated, and cleavage by *Hpa*II is blocked if the outer cytosine is hemi-methylated or the inner cytosine is methylated on both strands. If both cytosines are methylated, cleavage by both enzymes is blocked. Eight fluorescently labeled primer pairs were used for selective amplification (**Table S3**). Similar to AFLP, we scored the presence or absence of epigenetic fragments of 69 to 450 bp for each enzyme combination. Next, the epigenetic fragments were transformed into a binary character matrix. Four types of methylation can be defined: type 1 when a fragment is present in both *Msp*I and *Hpa*II reactions (1,1) or there is no methylation; type 2 when a fragment is present in *Msp*I but absent in *Hpa*II (1,0), indicating full methylation of internal cytosines; type 3 when a fragment is absent in *Msp*I, but present in *Hpa*II (0,1) indicating hemimethylation of the external cytosine and type 4 when there is no fragment in either *Msp*I or *Hpa*II reactions (0,0) due to either full methylation of cytosines or sequence polymorphism at the recognition site (Schulz et al., 2013). We pooled these methylation states into two categories: methylated (type 2 and type 3 coded as 1) and unmethylated (type 1 coded as 0) to create a new data matrix and used this matrix to compute epigenetic parameters. We treated type 4 as missing data, because the methylation state cannot be distinguished from sequence polymorphism (Foust et al., 2016).

### Meteorological Data

We collected meteorological data for 10 of the 11 natural-population regions (excluding TT) and the 6 cultivation regions from the National Climate Center in China (https://www.ncc-cma.net/cn/) for the period of October 2013 to May 2014 based on the annual planting time. The parameters were as follows: average (TEM_Avg, °C), lowest (TEM_Min, °C) and highest daily temperatures (TEM_Max, °C); average daily relative humidity (RHU_Avg, %); average atmospheric pressure (PRS_Avg, hpa); total daily sunshine time (SSH, hour); and total daily precipitation (PRE_Time, mm). We used the monthly means as weather parameters in subsequent calculations (**Table S4**).

### Analysis of Alkaloids

The major active components of interest in the rhizome of *C. yanhusuo* are alkaloids. Tetrahydropalmatine is the most important alkaloid in *C. yanhusuo*, and three other alkaloids, i.e., protopine, palmatinem, and berberine, are frequently used to assess the medicinal quality of *C. yanhusuo*. Therefore, we determined the contents of these four alkaloids in this study.

We mashed the dried rhizomes and passed them through a 40-mesh sieve. Next, 1.0 g of sample powder was ultrasonically extracted in 30 mL of 70% ethanol for 30 min and centrifuged for 1.5 min at 1000 r/min. The supernatant was filtered through a 0.45-μm membrane and used for high-performance liquid chromatography (HPLC) analysis. Chromatograms were generated on an Agilent 1100 series HPLC system (Agilent technologies, Palo Alto, CA USA). Chromatographic separation was performed on a Zorbax Extend C18 column using a gradient of A (acetonitrile) and B (0.06% triethylamine and 0.6% acetic acid), following the protocol of Zhao et al. (2013). After screening, a sufficiently large number of detectable peaks were visualized at a wavelength of 280 nm. The chromatographic peaks detected by HPLC were characterized by liquid chromatography–mass spectrometry. The mass spectra were acquired in the positive-ion mode using a Finnigan LCQDECA XP System (Thermo LC/MS Division, San Jose, CA, USA). The mass spectrometry detector (MSD) parameters were set according to Zhao et al. (2013). The contents of protopine, coptisine, tetrahydropalmatine, and palmatine were calculated using a calibration curve, which was plotted as peak area against the concentration determined from duplicate injections of six methanol-diluted working solutions of the reference compounds.

### Data Analysis

We evaluated both genetic and epigenetic diversity by calculating Shannon’s diversity index (*H*′) with GENALEX software (ver. 6.5; Peakall and Smouse, 2012). In addition, we used the “msap” package in R to calculate the frequency of each methylation type (Schulz et al., 2013).

Using the genetic data only, we performed the Bayesian clustering model implemented in STRUCTURE (ver. 2.3.4; Pritchard et al., 2000) to assess the genetic structure. This model infers population structure by clustering individual genotypes into a given number of populations (K) by minimizing deviation from Hardy-Weinberg equilibrium. The number of K was set to vary from 1 to 17, and 10 independent iterations were performed for each K. K was determined using the method of Evanno et al. (2005) based on the second-order rate of change of the likelihood (ΔK). Subsequently, we estimated genetic and epigenetic structure using principal coordinates analysis (PCoA) based on Nei’s distance matrix by GENALEX software. In addition, neighbor-joining (NJ) analysis (bootstraps = 1,000) was performed based on Nei’s distance using the PHYLIP Package (Felsenstein, 1993) for both AFLP and MSAP data. We calculated the pairwise epigenetic and genetic differentiation coefficient (*F*_ST_) (**Tables S5** and **S6**) and Nei’s distance (**Tables S7** and **S8**) with GENALEX software.

We further tested the assumption of population structure for both genetic and epigenetic loci using hierarchical analysis of molecular variance (AMOVA) to assess the overall differences among populations within natural compared to cultivated types (ARLEQUIN software ver. 3.5; Excoffier and Lischer, 2010). We also analyzed natural and cultivated populations separately to better understand how molecular variation was partitioned within and among populations of natural compared to cultivated plants. We tested significance with 10,000 permutations. We also calculated pairwise genetic differences among populations (*F*_ST_) in ARLEQUIN.

To explore patterns of spatial autocorrelation in natural and cultivated populations, we used simple linear regression of pairwise comparisons to calculate the correlations between genetic and epigenetic differentiation (*F*_ST_/1-*F*_ST_) and geographic distance (log-transformed) (Diniz-Filho et al., 2013) with the “lm” function in R. To estimate the relationship between genetic and epigenetic variation, we also analyzed the correlation between the pairwise genetic and epigenetic distances (*F*_ST_) and between the respective Shannon’s diversities (*H*’) with the “lm” function in R using the following formula: lm (x~y) where x = *F*_ST_ or *H*′ for AFLP, y = *F*_ST_ or *H*′ for MSAP.

To test the relationship between genetic, epigenetic, environmental, and phenotypic variation, we used distance-based redundancy analysis (dbRDA) with the “capscale” function of the VEGAN package in R (Oksanen et al., 2019). First, we evaluated the correlation between genetic and epigenetic variation separately for cultivated and natural populations using the following formula: capscale (x~y) where x = the Euclidean distance matrix for MSAP as the dependent variable, and y = the first three principal component analysis (PCA) axes of AFLP (AFLP_PC1, AFLP_PC2, AFLP_PC3) as predictors of epigenetic variation. We derived the PCs using the “principal” function of the psych package in R to run PCA with the following formula: principal (*r*, nfactors = 3) where *r* = the binary character matrix for AFLP. Then, we tested and quantified the overall contribution of environmental variables to epigenetic, genetic, and phenotypic divergence among populations in three separate dbRDA for natural populations and 3 separate dbRDA for cultivated populations using the formula: capscale (x~y) where x = the Euclidean distance matrix for AFLP or MSAP or alkaloids, y = meteorological factors (TEM_Avg, TEM_Min, TEM_Max, RHU_Avg, PRS_Avg, SSH, PRE_Time). Our environmental conditions were the seven meteorological factors and phenotypes were the contents of four alkaloids. Finally, we tested the correlations between alkaloid content and genetic or epigenetic variation in separate dbRDA of cultivated populations only using the following formula: capscale (x~y) where x = the Euclidean distance matrix for AFLP or MSAP across the six cultivated populations, and y = the content of four alkaloids (tetrahydropalmatine, protopine, palmatinem, berberine). Because we did not collect meteorological data from the TT natural-population region, we excluded TT from dbRDA analysis. To further assess the significance of the associations, we conducted permutation tests using the “envfit” function of the VEGAN package in R with 999 permutations.

## Results

### Genetic and Epigenetic Diversity

**Figure 2:**
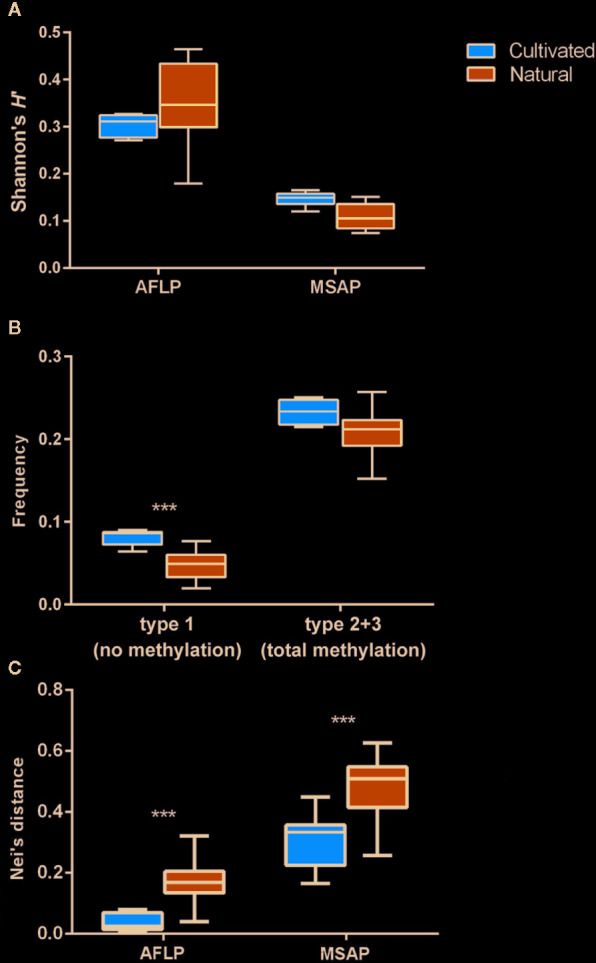
Magnitudes of genetic and epigenetic variation between natural and cultivated *C. yanhusuo* populations using methylation sensitive amplified polymorphism (MSAP) and amplified fragment length polymorphism (AFLP). **(A)** Shannon’s diversity (*H*′); **(B)** frequency of two types of methylation, including type 1 (no methylation), type 2+3 (total methylation); and **(C)** Nei’s distance. Boxplots indicate medians, 25th and 75th percentiles, minimum and maximum values. Significance between natural and cultivated populations in **(B, C)** based on Duncan tests with 10^4^ replications is indicated as: ^***^ *P* < 0.001.

The AFLP analysis of 313 individuals yielded 515 genetic loci, and the MSAP analysis resulted in 1337 epigenetic loci. We found that the mean genetic diversity was slightly higher in natural than in cultivated populations (*H*′_AFLP_: 0.3497 ± 0.0036 in natural compared to 0.3037 ± 0.0049 in cultivated; **Table 1**; **Figure 2A**), while epigenetic diversity was lower in natural populations compared to cultivated populations (*H*′_MSAP_: 0.1088 ± 0.0020 compared to 0.1470 ± 0.0029; **Table 1**; **Figure 2A**). Compared to natural populations, the frequency of unmethylated loci (type 1) was significantly higher in cultivated populations (**Figure 2B**; **Table S1**). The epigenetic diversities were generally lower than the genetic diversities in both the natural (*H*′_AFLP_: 0.3497 ± 0.0036; *H*′_MSAP_: 0.1088 ± 0.0020) and cultivated (*H*′_AFLP_: 0.3037 ± 0.0049; *H*′_MSAP_: 0.1470 ± 0.0029) populations (**Table 1**), but on average, epigenetic diversity was higher in cultivated populations. The mean pairwise genetic and epigenetic Nei’s distances were higher in the natural than in the cultivated populations (**Figure 2C**, **Tables S7** and **S8**).

### Genetic and Epigenetic Structure

**Figure 3:**
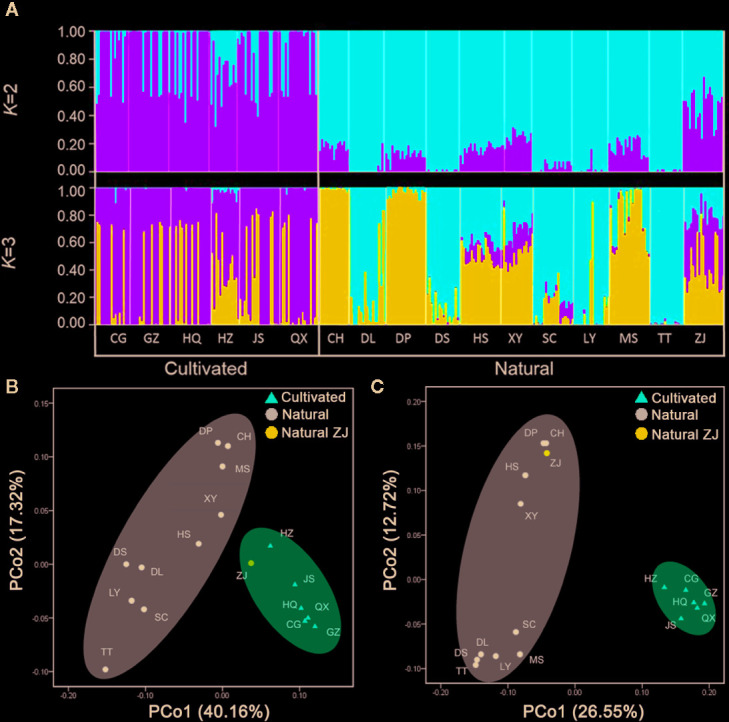
Genetic and epigenetic populations structure of *C. yanhusuo* using STRUCTURE and PCoA analysis. **(A)** Individual assignment to each cluster for K=2 to 3 based on genetic dat. Each individual is represented by a thin vertical line. Principal coordinates analysis (PCoA) among *C. yanhusuo* based on the AFLP profile **(B)** and MSAP profile **(C)**.

**Figure 4:**
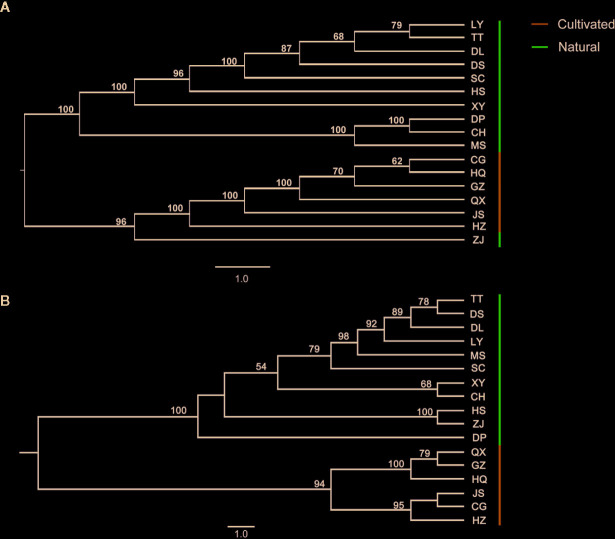
Results of the Neighbor-joining (NJ) analysis for natural and cultivated *C. yanhusuo* based on the Nei’s distance using **(A)** AFLP and **(B)** MSAP data.

Structure identified only two genetic groups: the ΔK criterion reached its nodal value for K = 2 (**Figure S1**), suggesting that the uppermost level of the genetic structure has two distinct clusters. The results for K = 2 and K = 3 are presented to illustrate the formation of populations (**Figure 3A**). At K = 2, all populations were divided into two clusters. Cluster I (green) was mainly present in the cultivated populations, whereas most individuals from the natural populations were assigned to cluster II (red), except for the ZJ population, which was assigned to both cluster I (0.54) and cluster II (0.46). At K = 3, cluster II was further separated into two clusters (red and blue), and the blue cluster was found in some individuals belonging to cultivated populations. The PCoA analysis showed that the first two principal coordinates account for 57.48% of the genetic variation of *C. yanhusuo* population, which mainly reflected the division between natural and cultivated populations, while the ZJ natural population nested within the cultivated cluster (**Figure 3B**). In the PCoA analysis for epigenetic data, the first two principal coordinates account for 39.27% of the epigenetic variation of *C. yanhusuo* populations, and natural and cultivated populations formed two clusters apart from each other (**Figure 3C**). In the NJ tree based on genetic data (**Figure 4A**), the cultivated and natural populations were grouped into two distinct clades, except ZJ, which clustered within the cultivated populations. In the NJ tree based on epigenetic data (**Figure 4B**), all of the natural populations formed a strongly supported clade (100% bootstrap support), whereas all cultivated populations clustered into the other clade (94% bootstrap support).

**Figure 5:**
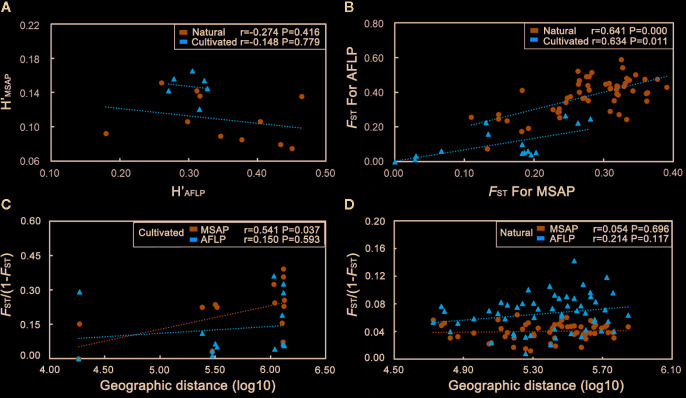
Correlations between epigenetic and genetic **(A)** diversity (*H*′) or **(B)** variation (*F*_ST_) in *C. yanhusuo* populations. Spatial autocorrelation pattern detected for epigenetic variation (*F*_ST_/1-*F*_ST_) in cultivated **(C)** and natural **(D)** populations. Geographic distance is log-transformed. r = correlation coefficient, P = significance of the correlation.

The results of the hierarchical AMOVA showed that 23.24% of the genetic variation and 20.89% of the epigenetic variation were explained by differences between cultivated and natural populations, while 25.12% of the genetic and 19.16% of the epigenetic variation was attributed to differences among populations within groups (**Table 2A**). Separate AMOVAs on natural and cultivated populations showed that more of the genetic variation (39.18%) than epigenetic variation (28.74%) was explained by differences among natural populations. On the contrary, less of the genetic variation (10.27%) in cultivated populations was explained by differences among populations then the amount of epigenetic variation (15.60%, **Table 2B**), indicating that most of the genetic and epigenetic variation in cultivated plants was found within each population. The genetic and epigenetic differences (*F*_ST_ 0.103 and 0.156) among cultivated populations were lower than those among natural populations (*F*_ST_ 0.392 and 0.287) (**Table 2**). We did not detect a correlation between pairwise comparisons of genetic (*H*′_AFLP_) and epigenetic diversity (*H*′_MSAP_) in either cultivated or natural populations (cultivated: r = −0.148, P = 0.779; natural: r = −0.274, P = 0.416; **Figure 5A**). However, we found that the genetic and epigenetic pairwise *F*_ST_ were significantly related in cultivated and natural populations (cultivated: r = 0.634, P = 0.011; natural: r = 0.641, P = 0.000; **Figure 5B**). We also found a spatial autocorrelation pattern for epigenetic variation in the cultivated populations (r = 0.541, P = 0.037; **Figure 5C**) but not in the natural populations (r = 0.054, P = 0.696; **Figure 5D**).

**Table 2:** (A) Three-level hierarchical analysis of epigenetic and genetic molecular variation (AMOVA) for 17 populations of *Corydalis yanhusuo* (B) Genetic and epigenetic AMOVA for natural and cultivated populations analyzed separately.

Source of variation	d.f.	Variance components	Percentage of variation	*F*-statistics
**A) Hierarchical AMOVA**				
**Genetic variation**				
Between cultivated and natural	1	28.20	23.24	*F*sc = 0.327
Among populations within cultivated/natural	15	30.49	25.12	*F*st = 0.484
Within populations	296	62.67	51.64	*F*ct = 0.232
				
**Epigenetic variation**				
Between cultivated and natural	1	53.56	20.89	*F*sc = 0.242
Among populations within cultivated/natural	15	49.11	19.16	*F*st = 0.401
Within populations	296	153.67	59.95	*F*ct = 0.209

**B) AMOVA**				
**Cultivated populations:**				
**Genetic variation**				
Among populations	5	6.42	10.27	*F*st = 0.103
Within populations	106	56.09	89.73	
**Epigenetic variation**				
Among populations	5	31.58	15.60	*F*st = 0.156
Within populations	106	170.80	84.40	
				
**Natural populations:**				
**Genetic variation**				
Among populations	10	42.74	39.18	*F*st = 0.392
Within populations	190	66.34	60.82	
**Epigenetic variation**				
Among populations	10	58.11	28.74	*F*st = 0.287
Within populations	190	144.10	71.26	

d.f., degrees of freedom; F-statistics: the proportion of variability among populations (Fst), among populations within groups (Fsc) and among groups (Fct).

### Relationships among Epigenetic, Genetic, Alkaloids, and Environmental Variation

**Figure 6:**
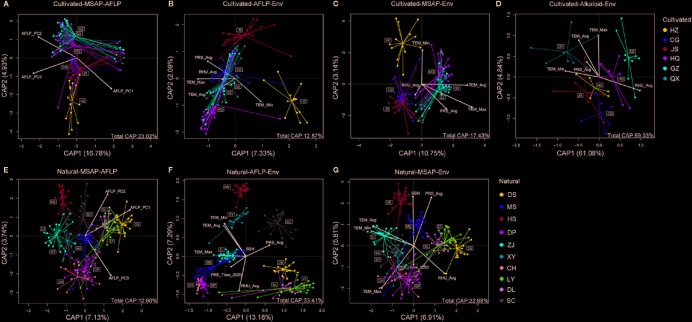
The relationships among epigenetic, genetic, alkaloids, and environmental variations using distance-based redundancy analyses (dbRDA). **(A)** epigenetic variation in cultivated populations of *C. yanhusuo* using genetic data as predictor; **(B)** genetic, **(C)** epigenetic, and **(D)** alkaloids variation in cultivated populations of *C. yanhusuo* using environmental factors as predictor; **(E)** the epigenetic variation in natural populations of *C. yanhusuo* using genetic data as predictor; **(F)** genetic and **(G)** epigenetic variation in cultivated populations of *C. yanhusuo* using environmental factors as predictor. For genetic data used as predictors of epigenetic variation, the first three PCA axes of the AFLP datasets were adopted. Environmental data include average (TEM_Avg), lowest (TEM_Min), and highest daily temperatures (TEM_Max), average daily relative humidity (RHU_Avg), average air pressure (PRS_Avg), total daily sunshine time (SSH) and total daily precipitation (PRE_Time). The percentages on the axes indicated the proportion of eigenvalues for each constrained axe, and the percentage in the bottom right corner of the graph indicated the proportion of eigenvalues for all constrained axes.

We analyzed the relationship between genetic, epigenetic, and environmental variation by separate dbRDA. We found that genetic variation explained 12.60% and 23.02% of the epigenetic variation in the natural and cultivated populations, respectively (**Figures 6A, E**). The environmental descriptors explained 33.41% of the genetic variation in natural populations, but only 12.87% of the genetic variation in cultivated populations (**Figures 6B, F**). Similarly, environmental factors explained 22.68% and 17.43% of the epigenetic variation in the natural and cultivated populations (**Figures 6C, G**). Both epigenetic and genetic variation were also correlated to almost every single climate variable in cultivated and natural populations (**Table S9**).

We detected alkaloid content in cultivated populations and found that the contents of four alkaloids differed significantly among the six cultivated populations (**Table 3**), even though genetic differentiation among the cultivated populations is limited. We further explore the relationships among epigenetic, genetic, and alkaloids variation by dbRDA, which suggested that alkaloid content was significantly correlated to both genetic (6.89%) and epigenetic (14.09%) variation in cultivated populations (**Figure S1**). All four alkaloids were significantly correlated with epigenetic variation and three (except protopine) were correlated with genetic variation (**Table 3**). Additionally, the environmental descriptors explained 69.03% of the variation in alkaloid content, and five environmental factors were significantly correlated with the alkaloid variation (the exception being PRS_Avg and SSH) (**Figure 6D**, **Table S9**).

**Table 3:** The contents of four alkaloids for 6 cultivated populations of *Corydalis yanhusuo* and results of correlating alkaloid variation to epigenetic and genetic variation.

		Protopine	Palmatine	Berberine	Tetrahydropalmatine
Alkaloid content (mg/g)	CG	0.3484 ± 0.0467 a	0.1289 ± 0.0317 a	0.0496 ± 0.0073 a	0.7543 ± 0.1038 a
	GZ	0.3415 ± 0.0626 c	0.2244 ± 0.0514 b	0.0773 ± 0.0146 a	0.4634 ± 0.0584 d
	HQ	0.4312 ± 0.0840 b	0.1402 ± 0.0285 c	0.0525 ± 0.0071 b	0.6117 ± 0.0980 c
	HZ	0.4468 ± 0.0648 c	0.1545 ± 0.0276 c	0.0574 ± 0.0037 b	0.7858 ± 0.1178 b
	JS	0.3836 ± 0.0575 b	0.2187 ± 0.0767 c	0.0948 ± 0.0322 b	0.8839 ± 0.1579 b
	QX	0.5475 ± 0.0578 bc	0.2837 ± 0.0552 b	0.0876 ± 0.0216 a	1.0124 ± 0.1945 bc
AFLP	r^2^	0.0125	0.4013^***^	0.4316^***^	0.1421^**^
MSAP	r^2^	0.1116^***^	0.3768^***^	0.3182^***^	0.0980^**^

Values marked by different letters indicate significant differences by Duncan test (P < 0.05). r^2^ represents the correlation coefficient between either AFLP or MSAP marker matrices and each alkaloid across all cultivated populations from the dbRDA. P stands for significance test of correlation which values are indicted as: ^**^P < 0.01; ^***^P < 0.001.

## Discussion

In this study, we investigated genetic and epigenetic variation among all 11 known natural populations of *C. yanhusuo* and compared these patterns of variation to 6 major cultivated populations. Despite evidence for a single origin of the cultivated plants (ZJ population), genetic diversity was only slightly higher in natural populations (*H*′_AFLP_: 0.3497 ± 0.0036) compared to cultivated populations (*H*′_AFLP_: 0.3037 ± 0.0049). However, genetic diversity is more evenly represented among populations in cultivation: only about 10% of the genetic variation was explained by differences among populations in cultivation compared to 38% in wild populations. The lower genetic differentiation for the cultivated plants is consistent with our hypothesis based on the differences in reproductive patterns between natural and cultivated populations of *C. yanhusuo*. In the wild, natural *C. yanhusuo* is mainly outcrossed by bee (Maloof, 2000; Qiu et al., 2009), which limits long-distance gene flow among natural populations. On the other hand, cultivated populations originated from a single source and have been propagated asexually so we expected lower genetic variation within and among populations. The hierarchical analysis showed that 23% of the overall genetic variation was due to differences between cultivated and wild populations suggesting that a large portion of the genetic variation within the cultivated populations was not present in the natural populations. This unexplained genetic variation in cultivated plants could be partly due to somatic mutation since several studies have reported that high rates of somatic mutation may allow asexual species to maintain abundant genetic variation and adapt to changing environmental conditions (Lynch, 1984; Gill et al., 1995; Schoen and Schultz, 2019). The modular nature of clonal plants allows for detrimental mutations to be terminated in specific cell lines while benign or beneficial mutations can be maintained (reviewed in Schoen and Schultz, 2019). However, we cannot exclude the possibility that cultivars were also introduced from other areas. Understanding the sources of genetic variation in the cultivated populations will require more detailed genetic investigations.

In contrast to the patterns of genetic variation, we detected that epigenetic variation among cultivated populations (*H*′_MSAP_: 0.1470 ± 0.0029) exceeded that of natural populations (*H*′_MSAP_: 0.1088 ± 0.0020). We predicted that epigenetic differentiation would also be higher in cultivated than natural populations but we found the opposite (*F*_ST_ = 0.156 compared to *F*_ST_ = 0.287). These results suggest higher levels of epigenetic diversity that are more evenly represented among cultivated populations. Therefore, it appears that cultivated populations have relatively less genetic than epigenetic differentiation, while the opposite is true for natural populations. Combined, these results suggest that processes involved in cultivation could have an influence on the diversity and distribution of genetic and epigenetic modifications.

Understanding the causes and consequences of natural epigenetic variation is not straightforward, considering that epigenetic variation is affected by many factors, including genetic and environmental variation (Richards et al., 2017). In our study, we showed that epigenetic variation was significantly correlated to genetic variation with pairwise *F*_ST_ comparisons and a significant association in dbRDA. However, more of the epigenetic diversity was explained by genetic diversity in the cultivated (23.02%) than in the natural (12.60%) populations. We also found that more of the genetic (33.41% compared to 12.87%) and epigenetic (22.68% compared to 17.43%) variation was explained by climatic factors in natural populations than in cultivated populations of *C. yanhusuo*. This could be partly due to the greater number of natural populations exposed to a wider range of environments. On the other hand, epigenetic modification can mediate phenotypic plasticity (Herman et al., 2014; Herman and Sultan, 2016; Smith et al., 2016; Kilvitis et al., 2017; Richards et al., 2017; Banta and Richards, 2018), which can be important for response of domestication traits to novel or less than optimal conditions (Piperno, 2017), and impact which individuals are selected for cultivation. Selecting individuals that can tolerate a variety of new environments could be selecting for individuals with the ability to express more epigenetic variation (Herman et al., 2014; Herman and Sultan, 2016; Richards et al., 2017; Banta and Richards, 2018). In fact, Hauben et al. (2009) were able to artificially select on epigenetic variation to create developmental differences among individuals from an isogenic canola population. More recently, Shen et al. (2018) found 5,412 differentially methylated regions (DMRs) that corresponded with soybean domestication and improvement. Several DMRs were associated with genes involved in carbohydrate metabolism and were not associated with genetic variation, indicating that functional changes in methylation may be independent from genetic variation.

In our study, we showed that the alkaloid content varied among cultivated populations, and was more highly correlated with epigenetic (14.09%) than genetic (6.89%) variation. This could result from selection of genotypes with a greater potential for epigenetic diversity during the cultivation process, or from the clonal propagation which is thought to limit methylation reprogramming (Richards et al., 2012; Dodd and Douhovnikoff, 2016; González et al., 2018). However, epigenetic variation was not as strongly explained by the climate factors we analyzed in the cultivated plants as in the natural plants. The increased epigenetic variation in cultivated plants could be a response to an unmeasured environmental factor or to the process of domestication. Climatic variation was the strongest predictor of alkaloid content (69%), suggesting processes other than DNA methylation are involved in translating environmental variation into phenotypic variation (e.g., processes that contribute to transcription variation; Sauvage et al., 2017; Liu et al., 2019), or that our methods have not captured the causal methylation patterns (Richards et al., 2017; Paun et al., 2019). Still, these results indicate that persistent epigenetic variation could be contributing to differences in phenotypes and genetic background is less important. However, our study cannot specifically address the possibility that the selected cultivars were those that were more prone to increase epigenetic variation or if the cultivars under selection have increased levels of these alkaloids compared to natural populations. Further studies are required to evaluate the contribution of genetic, epigenetic, and environmental variation to the production of these alkaloids, and response to environmental challenges in asexual crop plants (Verhoeven and Preite, 2014; Dodd and Douhovnikoff, 2016).

## Conclusion

We used both natural and cultivated populations to identify slightly higher levels of genetic variation in natural than in cultivated populations and the opposite pattern for epigenetic variation. Epigenetic variation was significantly correlated to genetic variation but more strongly so in cultivated populations. We found higher epigenetic but lower genetic differentiation in cultivated populations while the reverse was true in natural populations. Such differences between cultivated and wild populations might be caused by the process of cultivation and/or the different modes of reproductive between the two types of *C. yanhusuo*. Epigenetic variation explained more of the variation in alkaloid content than genetic variation, suggesting that epigenetic variation could be particularly important in the process of domestication and provide an important source of phenotypic variation in cultivated (asexual) *C. yanhusuo*.

## Data Availability Statement

The datasets generated for this study are available on request to the corresponding authors.

## Author Contributions

CC, ZS, and CF planned and designed the research. ZZ, YB, and HZ performed AFLP and MSAP experiments. YZ and JL performed alkaloids analysis. YC conducted fieldwork. CC and ZZ analyzed data. CC and CR wrote the manuscript.

## Funding

This research was supported by the National Science Foundation of China (31770404), the Fundamental Research Funds for the Central Universities (KYZ201739), the National Key Research and Development Program of China (2016YFD0800803), the Key Breeding Project of Zhejiang Province (2012C12912, 2016C02058), China’s Postdoctoral Science Foundation (2014M560428, 2016T90468), the China Agriculture Research System (CARS-10-B24) and Deutscher Akademische Austauschdienst (DAAD; MOPGA Project ID 306055 to CR).

## Conflict of Interest

The authors declare that the research was conducted in the absence of any commercial or financial relationships that could be construed as a potential conflict of interest.

## References

[B1] Allaby R. G. Ware R. L. Kistler L. (2019). A re-evaluation of the domestication bottleneck from archaeogenomic evidence. Evol. Appl. 12, 29–37. 10.1111/eva.12680 30622633 PMC6304682

[B2] Alonso C. Balao F. Bazaga P. Pérez R. (2016). Epigenetic contribution to successful polyploidizations: variation in global cytosine methylation along an extensive ploidy series in *Dianthus broteri* (Caryophyllaceae). New Phytol. 212, 571–576. 10.1111/nph.14138 27483440

[B3] Banta J. B. Richards C. L. (2018). Quantitative epigenetics and evolution. Heredity 121, 210–224. 10.1038/s41437-018-0114-x 29980793 PMC6082842

[B4] Becker C. Hagmann J. Müller J. Koenig D. Stegle O. Borgwardt K. (2011). Spontaneous epigenetic variation in the *Arabidopsis thaliana* methylome. Nature 480, 245–249. 10.1038/nature10555 22057020

[B5] Berger J. D. Buirchell B. J. Luckett D. J. Nelson M. N. (2012). Domestication bottlenecks limit genetic diversity and constrain adaptation in narrow-leafed lupin (*Lupinus angustifolius* L.). Theor. Appl. Genet. 124, 637–652. 10.1007/s00122-011-1736-z 22069118

[B6] Caicedo A. L. Williamson S. H. Hernandez R. D. Boyko A. Fledel-Alon A. York T. L. (2007). Genome-wide patterns of nucleotide polymorphism in domesticated rice. PloS Genet. 3, e163. 10.1371/journal.pgen.0030163 PMC1994709 17907810

[B7] Darwin C. (1868). The variation of plants and animals under domestication (London: J. Murray).

[B8] Denham T. Barton H. Castillo C. Crowther A. Dotte-Sarout E. Florin A. (2020). The domestication syndrome in vegetatively-propagated field crops. Ann. Bot. 3, 581–597. 10.1093/aob/mcz212 PMC7102979 31903489

[B9] Diniz-Filho J. A. Soares T. N. Lima J. S. Dobrovolski R. Landeiro V. L. Telles M. P. D. C. (2013). Mantel test in population genetics. Genet. Mol. Biol. 36, 475–485. 10.1590/S1415-47572013000400002 24385847 PMC3873175

[B10] Dodd R. S. Douhovnikoff V. (2016). Adjusting to global change through clonal growth and epigenetic variation. Front. Ecol. Environ. 4, 86. 10.3389/fevo.2016.00086

[B11] Dubin M. J. Zhang P. Meng D. Remigereau M. S. Osborne E. J. Casale F. P. (2015). DNA methylation in *Arabidopsis* has a genetic basis and shows evidence of local adaptation. Elife 4, e05255. 10.7554/eLife.05255 25939354 PMC4413256

[B12] Evanno G. Regnaut S. Goudet J. (2005). Detecting the number of clusters of individuals using the software STRUCTURE: a simulation study. Mol. Ecol. 14, 2611–2620. 10.1111/j.1365-294X.2005.02553.x 15969739

[B13] Excoffier L. Lischer H. E. L. (2010). Arlequin suite ver 3.5: a new series of programs to perform population genetics analyses under Linux and Windows. Mol. Ecol. Res. 10, 564–567. 10.1111/j.1755-0998.2010.02847.x 21565059

[B14] Felsenstein J. (1993). PHYLIP (phylogenetic inference package) version 3.6. Department of Genetics, University of Washington. Seattle 63, 188–192.

[B15] Feng S. Jacobsen S. E. (2011). Epigenetic modifications in plants: an evolutionary perspective. Curr. Opin. Plant Biol. 14, 179–186. 10.1016/j.pbi.2010.12.002 21233005 PMC3097131

[B16] Foust C. M. Preite V. Schrey A. W. Alvarez M. Robertson M. H. Verhoeven K. J. F. (2016). Genetic and epigenetic differences associated with environmental gradients in replicate populations of two salt marsh perennials. Mol. Ecol. 25, 1639–1652. 10.1111/mec.13522 26880043

[B17] Fuller D. Q. Denhamb T. Arroyo-Kalina M. Lucas L. Stevens C. J. Qin L. (2011). Convergent evolution and parallelism in plant domestication revealed by an expanding archaeological record. Proc. Natl. Acad. Sci. 111, 6147–6152. 10.1073/pnas.1308937110 PMC4035951 24753577

[B18] Gallusci P. Dai Z. Génard M. Gauffretau A. Leblanc-Fournier N. Richard-Molard C. (2017). Epigenetics for plant improvement: current knowledge and modeling avenues. Trends Plant Sci. 22, 610–623. 10.1016/j.tplants.2017.04.009 28587758

[B19] Gehring M. (2019). Epigenetic dynamics during flowering plant reproduction: evidence for reprogramming? New Phytol. 224, 91–96. 10.1111/nph.15856 31002174 PMC6711810

[B20] Gill D. E. Chao L. Perkins S. L. Wolf J. B. (1995). Genetic mosaicism in plants and clonal animals. Annu. Rev. Ecol. Syst. 26, 423–444. 10.1146/annurev.es.26.110195.002231

[B21] González A. P. R. Preite V. Verhoeven K. J. F. Latzel V. (2018). Transgenerational effects and epigenetic memory in the clonal plant *Trifolium repens* . Front. Plant Sci. 9, 1677. 10.3389/fpls.2018.01677 30524458 PMC6256281

[B22] Gugger P. F. Fitz-Gibbon S. Pellegrini M. Sork V. L. (2016). Species-wide patterns of DNA methylation variation in *Quercus lobata* and their association with climate gradients. Mol. Ecol. 25, 1665–1680. 10.1111/mec.13563 26833902

[B23] Hauben M. Haesendonckx B. Standaert E. Van Der Kelen K. Azmi A. Akpo H. (2009). Energy use efficiency is characterized by an epigenetic component that can be directed through artificial selection to increase yield. Proc. Natl. Acad. Sci. 106, 20109–20114. 10.1073/pnas.0908755106 19897729 PMC2774259

[B24] Heard E. Martienssen R. A. (2014). Transgenerational epigenetic inheritance: myths and mechanisms. Cell 157, 95–109. 10.1016/j.cell.2014.02.045 24679529 PMC4020004

[B25] Herman J. J. Sultan S. E. (2016). DNA methylation mediates genetic variation for adaptive transgenerational plasticity. Proc. Biol. Sci. 283, 20160988. 10.1098/rspb.2016.0988 27629032 PMC5031651

[B26] Herman J. J. Spencer H. G. Donohue K. Sultan S. E. (2014). How stable ‘should’ epigenetic modifications be? Insights from adaptive plasticity and bet hedging. Evolution 68, 632–643. 10.1111/evo.12324 24274594

[B27] Herrera C. M. Bazaga P. (2010). Epigenetic differentiation and relationship to adaptive genetic divergence in discrete populations of the violet *Viola cazorlensis* . New Phytol. 187, 867–876. 10.1111/j.1469-8137.2010.03298.x 20497347

[B28] Herrera C. M. Medrano M. Bazaga P. (2017). Comparative epigenetic and genetic spatial structure of the perennial herb *Helleborus foetidus* : Isolation by environment, isolation by distance, and functional trait divergence. Am. J. Bot. 104 (8), 1195–1204. 10.3732/ajb.1700162 28814406

[B29] Hufford M. B. Xu X. van Heerwaarden J. Pyhajarvi T. Chia J. M. Cartwright R. A. (2012). Comparative population genomics of maize domestication and improvement. Nat. Genet. 44, 808–811. 10.1038/ng.2309 22660546 PMC5531767

[B30] Hyten D. L. Song Q. J. Zhu Y. L. Choi I. Y. Nelson R. L. Costa J. M. (2006). Impacts of genetic bottlenecks on soybean genome diversity. Proc. Natl. Acad. Sci. 103, 16666–16671. 10.1073/pnas.0604379103 17068128 PMC1624862

[B31] Ji L. Neumann D. A. Schmitz R. J. (2015). Crop epigenomics: identifying, unlocking, and harnessing cryptic variation in crop genomes. Mol. Plant 8, 860–870. 10.1016/j.molp.2015.01.021 25638564 PMC5121661

[B32] Jullien P. E. Berger F. (2010). DNA methylation reprogramming during plant sexual reproduction? Trends Genet. 26, 394–399. 10.1016/j.tig.2010.06.001 20609490

[B33] Kilvitis H. J. Hanson H. Schrey A. W. Martin L. B. (2017). Epigenetic potential as a mechanism of phenotypic plasticity in vertebrate range expansions. Integr. Comp. Biol. 57, 385–395. 10.1093/icb/icx082 28859411

[B34] Li X. Zhu J. Hu F. Ge S. Ye M. Xiang H. (2012). Single-base resolution maps of cultivated and wild rice methylomes and regulatory roles of DNA methylation in plant gene expression. BMC Genomics 13, 300. 10.1186/1471-2164-13-300 22747568 PMC3447678

[B35] Li Q. Eichten S. R. Hermanson P. J. Zaunbrecher V. M. Song J. Wendt J. (2014). Genetic perturbation of the maize methylome. Plant Cell. 26, 4602–4616. 10.1105/tpc.114.133140 25527708 PMC4311211

[B36] Liebl A. L. Schrey A. W. Andrew S. C. Sheldon E. L. Griffith S. C. (2015). Invasion genetics: lessons from a ubiquitous bird, the house sparrow *Passer* domesticus. Curr. Zool 61, 465–476. 10.1093/czoolo/61.3.465

[B37] Lin T. Zhu G. T. Zhang J. H. Xu X. Y. Yu Q. H. Zheng Z. (2014). Genomic analyses provide insights into the history of tomato breeding. Nat. Genet. 46, 1220–1226. 10.1038/ng.3117 25305757

[B38] Lira-Medeiros C. F. Parisod C. Fernandes R. A. Mata C. S. Cardoso M. A. Ferreira P. C. G. (2010). Epigenetic variation in mangrove plants occurring in contrasting natural environment. PloS One 5, e10326. 10.1371/journal.pone.0010326 20436669 PMC2859934

[B39] Liu W. Kang L. Xu Q. Tao C. Yan J. Sang T. (2019). Increased expression diversity buffers the loss of adaptive potential caused by reduction of genetic diversity in new unfavourable environments. Biol. Lett. 15, 20180583. 10.1098/rsbl.2018.0583 30958214 PMC6371898

[B40] Lu Y. Zhou D. Zhao Y. (2020). Understanding epigenomics based on the rice model. Theor. Appl. Genet. 133, 1345–1363. 10.1007/s00122-019-03518-7 31897514

[B41] Lynch M. (1984). Destabilizing hybridization, general-purpose genotypes and geographic parthenogenesis. Q. Rev. Biol. 59, 257–290. 10.1086/413902

[B42] Maloof J. E. (2000). Reproductive biology of a North American subalpine plant: *Corydalis caseana* A. Gray ssp. *Brandegei* (S. Watson) GB Ownbey. Plant Spec. Biol. 15, 281–288. 10.1111/j.1442-1984.2000.00047.x

[B43] Meyer R. S. Purugganan M. D. (2013). Evolution of crop species: genetics of domestication and diversification. Nat. Rev. Genet. 14, 840–852. 10.1038/nrg3605 24240513

[B44] Nicotra A. B. Atkin O. K. Bonser S. P. Davidson A. M. Finnegan E. J. Mathesius U. (2010). Plant phenotypic plasticity in a changing climate. Trends Plant Sci. 15, 684–692. 10.1016/j.tplants.2010.09.008 20970368

[B45] Oksanen J. Blanchet F. G. Friendly M. Kindt R. Legendre P. McGlinn D. (2019). Vegan: community ecology package. R package v.2.5-6. Available online at: http://CRAN.R-project.org/package=vegan.

[B46] Paun O. Verhoeven K. J. F. Richards C. L. (2019). Opportunities and limitations of reduced representation bisulfite sequencing in ecological epigenomics. New Phytol. 221, 738–742. 10.1111/nph.15388 30121954 PMC6504643

[B47] Peakall R. Smouse P. E. (2012). GenAlEx 6.5: genetic analysis in Excel. Population genetic software for teaching and research-an update. Bioinformatics 28, 2537–2539. 10.1093/bioinformatics/bts460 22820204 PMC3463245

[B48] Pharmacopoeia Committee of P. R. China (2010). Pharmacopoeia of People’s Republic of China (Beijing: Chemical Industry Publishers).

[B49] Piperno D. R. (2017). Assessing elements of an extended evolutionary synthesis for plant domestication and agricultural origin research. Proc. Natl. Acad. Sci. 25, 6429–6437. 10.1073/pnas.1703658114 PMC5488947 28576881

[B50] Pritchard J. K. Stephens M. Donnelly P. (2000). Inference of population structure using multilocus genotype data. Genetics 155, 945–959. 10835412 10.1093/genetics/155.2.945 PMC1461096

[B51] Purugganan M. D. Fuller D. Q. (2009). The nature of selection during plant domestication. Nature 457, 843–848. 10.1038/nature07895 19212403

[B52] Qiu Y. X. Zong M. Yao Y. Chen B. L. Zhou X. L. Fu C. X. (2009). Genetic variation in wild and cultivated *rhizoma corydalis* revealed by ISSRs markers. Planta Med. 75, 94–98. 10.1055/s-0028-1088365 19034828

[B53] Reyna-López G. E. Simpson J. Ruiz-Herrera J. (1997). Differences in DNA methylation patterns are detectable during the dimorphic transition of fungi by amplification of restriction polymorphisms. Mol. Gen. Genet. 253, 703–710. 10.1007/s004380050374 9079881

[B54] Richards C. L. Schrey A. W. Pigliucci M. (2012). Invasion of diverse habitats by few Japanese knotweed genotypes is correlated with epigenetic differentiation. Ecol. Lett. 15, 1016–1025. 10.1111/j.1461-0248.2012.01824.x 22731923

[B55] Richards C. L. Alonso C. Becker C. Bossdorf O. Bucher E. Colomé-Tatché M. (2017). Ecological plant epigenetics: Evidence from model and non-model species, and the way forward. Ecol. Lett. 20, 1576–1590. 10.1111/ele.12858 29027325

[B56] Sauvage C. Rau A. Aichholz C. Chadoeuf J. Sarah G. Ruiz M. (2017). Domestication rewired gene expression and nucleotide diversity patterns in tomato. Plant J. 91, 631–645. 10.1111/tpj.13592 28488328

[B57] Schoen D. J. Schultz S. T. (2019). Somatic Mutation and Evolution in Plants. Annu. Rev. Ecol. Evol. Syst. 50, 49–73. 10.1146/annurev-ecolsys-110218-024955

[B58] Schulz B. Eckstein R. L. Durka W. (2013). Scoring and analysis of methylation-sensitive amplification polymorphisms for epigenetic population studies. Mol. Ecol. Res. 13, 642–653. 10.1111/1755-0998.12100 23617735

[B59] Schulz B. Eckstein R. L. Durka W. (2014). Epigenetic variation reflects dynamic habitat conditions in a rare floodplain herb. Mol. Ecol. 23, 3523–3537. 10.1111/mec.12835 24943730

[B60] Shen Y. Zhang J. Liu Y. Liu S. Liu Z. Duan Z. (2018). DNA methylation footprints during soybean domestication and improvement. Genome Biol. 19, 128. 10.1186/s13059-018-1516-z 30201012 PMC6130073

[B61] Shi D. Zhuang K. Xia Y. Zhu C. Chen C. Shen Z. (2017). *Hydrilla verticillata* employs two different ways to affect DNA methylation under excess copper stress. Aquat. Toxicol. 193, 97–104. 10.1016/j.aquatox.2017.10.007 29053963

[B62] Smith T. A. Martin M. D. Nguyen M. Mendelson T. C. (2016). Epigenetic divergence as a potential first step in darter speciation. Mol. Ecol. 25, 1883–1894. 10.1111/mec.13561 26837057

[B63] Tricker P. J. (2015). Transgenerational inheritance or resetting of stress-induced epigenetic modifications: two sides of the same coin. Front. Plant Sci. 6, 699. 10.3389/fpls.2015.00699 26442015 PMC4561384

[B64] Verde I. Abbott A. G. Scalabrin S. Jung S. Shu S. Marroni F. (2013). The high-quality draft genome of peach (*Prunus persica*) identifies unique patterns of genetic diversity, domestication and genome evolution. Nat. Genet. 45, 487–494. 10.1038/ng.2586 23525075

[B65] Verhoeven K. J. Preite V. (2014). Epigenetic variation in asexually reproducing organisms. Evolution 68, 644–655. 10.1111/evo.12320 24274255

[B66] Vos P. Hogers R. Bleeker M. Reijans M. van de Lee T. Hornes M. (1995). AFLP: a new technique for DNA fingerprinting. Nucleic Acids Res. 23, 4407–4414. 10.1093/nar/23.21.4407 7501463 PMC307397

[B67] Wibowo A. Becker C. Marconi G. Durr J. Price J. Hagmann J. (2016). Hyperosmotic stress memory in Arabidopsis is mediated by distinct epigenetically labile sites in the genome and is restricted in the male germline by DNA glycosylase activity. Elife 5, e13546. 10.7554/eLife.13546 27242129 PMC4887212

[B68] Wibowo A. Becker C. Durr J. Price J. Spaepen S. Hilton S. (2018). Partial maintenance of organ-specific epigenetic marks during plant asexual reproduction leads to heritable phenotypic variation. Proc. Natl. Acad. Sci. 115, E9145–E9152. 10.1073/pnas.1805371115 30201727 PMC6166847

[B69] Xie H. J. Li A. H. Liu A. D. Dai W. M. He J. Y. Lin S. (2015). ICE1 demethylation drives the range expansion of a plant invader through cold tolerance divergence. Mol. Ecol. 24, 835–850. 10.1111/mec.13067 25581031

[B70] Xu Z. X. Wei J. H. (1994). The chromosome numbers and their relationships of several medicinal species in *Corydalis* . China J. Chin. Mater. Med. 19, 82–83. (in Chinese).

[B71] Zhao Y. P. Li J. H. Yang S. T. Fan J. Fu C. X. (2013). Effects of postharvest processing and geographical source on phytochemical variation of *corydalis rhizoma* . Chin. Herb. Med. 5, 151–157. 10.3969/j.issn.1674-6348.2013.02.013

[B72] Zhou Z. Jiang Y. Wang Z. Gou Z. Lyu J. Li W. (2015). Resequencing 302 wild and cultivated accessions identifies genes related to domestication and improvement in soybean. Nat. Biotechnol. 33, 408–414. 10.1038/nbt.3096 25643055

