# Supplementary material for: Comparisons of Natural and Cultivated Populations of Corydalis yanhusuo Indicate Divergent Patterns of Genetic and Epigenetic Variation
Source: Front Plant Sci. 2020 Jul 3;11:985. doi: 10.3389/fpls.2020.00985 (PMC7347962; doi:10.3389/fpls.2020.00985)
Supplement: Supplementary file 1 [file DataSheet_1.docx]

Supplementary Material

# Supplementary Figures and Tables

## Supplementary Figures


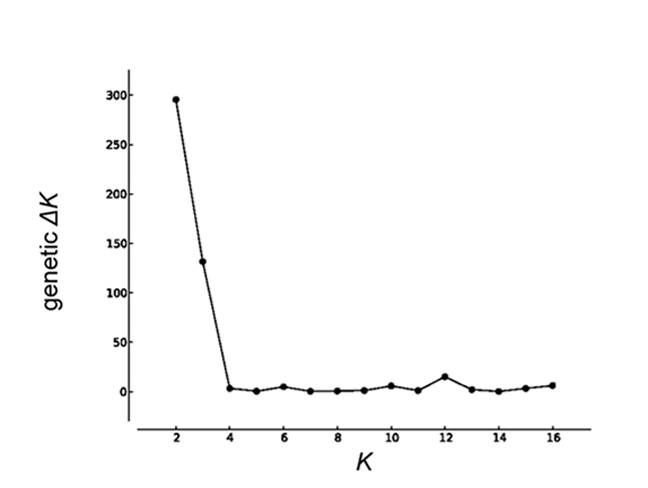


**Figure S1** The ΔK plot in STURCTURE analysis. ΔK was calculated as Evanno et al. (2005) for each K by admixture model.


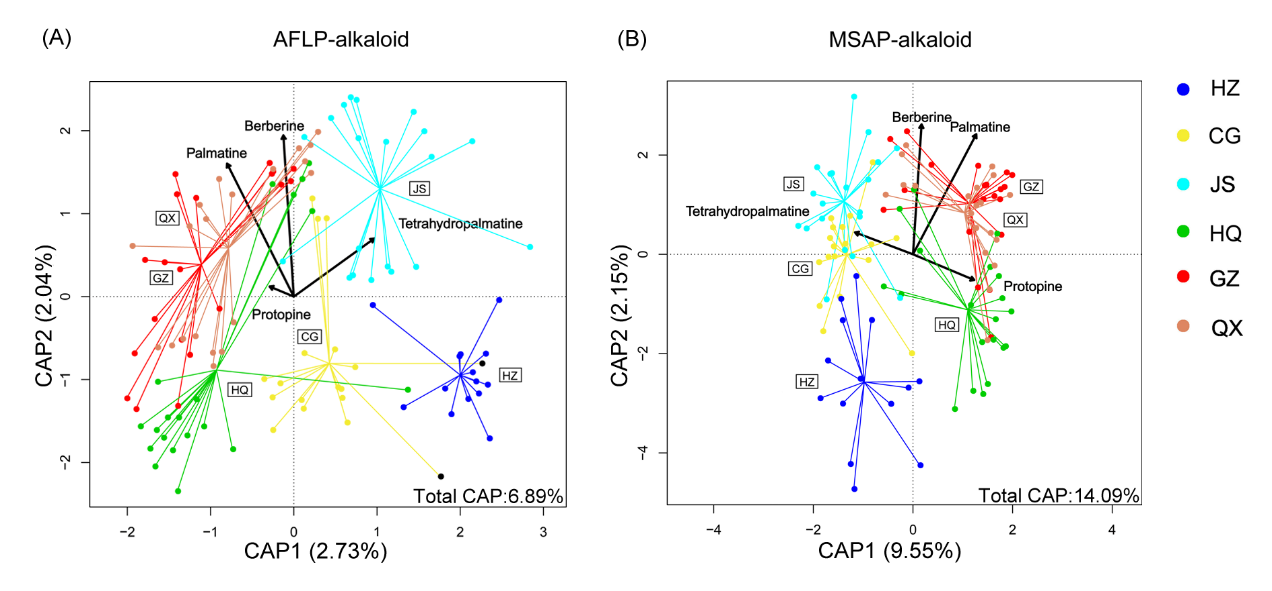


**Figure S2** Distance-based redundancy analyses (dbRDA) for genetic (A) and epigenetic (B) variation in cultivated *Corydalis yanhusuo* populations using the content of four alkaloids (tetrahydropalmatine, protopine, palmatinem and berberine) as predictor. The percentages on the axes indicated the proportion of eigenvalues for each constrained axe, and the percentage in the bottom right corner of the graph indicated the proportion of eigenvalues for all constrained axes.

## Supplementary Tables

**Table S1** Frequency of five types of methylation across the 17 populations of *Corydalis yanhusuo*

| Population |  | | MSAP (frequency of types) | | | | | | | | | |  |
| --- | --- | --- | --- | --- | --- | --- | --- | --- | --- | --- | --- | --- | --- |
|  |  | | Type 1 | | Type 2 | | Type 3 | | Type 4 | | Type 2+3 | |  |
| Cultivated |  | |  | |  | |  | |  | |  | |  |
| CG |  | | 0.0872 | | 0.1411 | | 0.0863 | | 0.6854 | | 0.2274 | |  |
| GZ |  | | 0.087 | | 0.1594 | | 0.0872 | | 0.6664 | | 0.2466 | |  |
| HQ |  | | 0.0755 | | 0.1366 | | 0.1034 | | 0.6845 | | 0.2400 | |  |
| HZ |  | | 0.0643 | | 0.1164 | | 0.0981 | | 0.7212 | | 0.2145 | |  |
| JS |  | | 0.0903 | | 0.1249 | | 0.0941 | | 0.6907 | | 0.2190 | |  |
| QX |  | | 0.0853 | | 0.1624 | | 0.0885 | | 0.6668 | | 0.2509 | |  |
| Natural | |  | |  | |  | |  | |  | |  | |
| CH |  | | 0.0197 | | 0.0734 | | 0.0789 | | 0.828 | | 0.1523 | |  |
| DL |  | | 0.0364 | | 0.1374 | | 0.0795 | | 0.7467 | | 0.2169 | |  |
| DP |  | | 0.0325 | | 0.0772 | | 0.0805 | | 0.8098 | | 0.1577 | |  |
| DS |  | | 0.0579 | | 0.131 | | 0.0849 | | 0.7262 | | 0.2159 | |  |
| HS |  | | 0.0768 | | 0.106 | | 0.0863 | | 0.731 | | 0.1923 | |  |
| XY |  | | 0.033 | | 0.1376 | | 0.0941 | | 0.7353 | | 0.2317 | |  |
| SC |  | | 0.0393 | | 0.1166 | | 0.0927 | | 0.7515 | | 0.2093 | |  |
| LY |  | | 0.0623 | | 0.1464 | | 0.077 | | 0.7142 | | 0.2234 | |  |
| MS |  | | 0.0602 | | 0.1611 | | 0.0963 | | 0.6824 | | 0.2574 | |  |
| TT |  | | 0.0492 | | 0.1282 | | 0.084 | | 0.7387 | | 0.2122 | |  |
| ZJ |  | | 0.0555 | | 0.104 | | 0.089 | | 0.7515 | | 0.1930 | |  |

Note: Frequency of five types of methylation in each population (%); type 1, no methylation; type 2, full methylation of internal cytosines; type 3, hemimethylation of the external cytosine; type 4, either full methylation of cytosines or sequence polymorphism at the recognition site; type 2+3, total methylation, the summation of full methylation and hemi-methylation.

**Table S2** Sequences of adapters and primers used for AFLP analysis. Primer pairs used for AFLP selective amplifications: E14*M34/ E14*M42/ E14*M43/ E21*M34/ E21*M43/ E34*M31/ E44*M31/ E44*M32.

| Primers/adapters | Oligonucleotide sequences (5′–3′) | |
| --- | --- | --- |
| *EcoR* I Adapters |  | |
| E1 | CTC GTA GAC TGC GTA CC | |
| E2 | AAT TGG TAC GCA GTC TAC | |
| *Mse* I Adapters |  | |
| M1 | GAC GAT GAG TCC TGA G | |
| M2 | TAC TCA GGA CTC AT | |
| Preselective primers |  | |
| E01 | GAC TGC GTA CCA ATT CA | |
| M03 | GAT GAG TCC TGA GTA AC | |
| *EcoR* I Selective amplification primers | |  |
| *Eco*R I_AAG E14 | GAC TGC GTA CCA ATT CAAG | |
| *Eco*R I_ATA E21 | GAC TGC GTA CCA ATT CATA | |
| *Eco*R I_ACG E34 | GAC TGC GTA CCA ATT CACG | |
| *Eco*R I_AGG E44 | GAC TGC GTA CCA ATT CAGG | |
| *Mse* I Selective amplification primers |  | |
| *Mse* I _CCA M31 | GAT GAG TCC TGA GTA ACCA | |
| *Mse* I _CCT M32 | GAT GAG TCC TGA GTA ACCT | |
| *Mse* I _CCG M34 | GAT GAG TCC TGA GTA ACCG | |
| *Mse* I _CGT M42 | GAT GAG TCC TGA GTA ACGT | |
| *Mse* I _CGC M43 | GAT GAG TCC TGA GTA ACGC | |

**Table S3** Sequences of adapters and primers used for MSAP analysis. Primer pairs used for MSAP selective amplifications: E41*HMTCGA/ E41*HMTGC/ E44*HMTGC/ E42*HMTCCA/ E21*HMTCA/ E34*HMTCA/ E21*HMTCGA/ E33*HMTCGA.

| Primers/adapters | Oligonucleotide sequences (5′–3′) |
| --- | --- |
| *EcoR* I Adapters |  |
| E1 | CTC GTA GAC TGC GTA CC |
| E2 | AAT TGG TAC GCA GTC TAC |
| *Hpa* II/*Msp* I Adapters |  |
| HM1 | GAT CAT GAG TCC TGC T |
| HM2 | CGA GCA GGA CTC ATG A |
| Preselective primers |  |
| E01 | GAC TGC GTA CCA ATT CA |
| HM0 | ATC ATG AGT CCT GCT CGG |
| *EcoR* I Selective amplification primers |  |
| *Eco*R I_ATA E21 | GAC TGC GTA CCA ATT CATA |
| *Eco*R I_TCC E33 | GAC TGC GTA CCA ATT CTCC |
| *Eco*R I_ACG E34 | GAC TGC GTA CCA ATT CACG |
| *Eco*R I_AGA E41 | GAC TGC GTA CCA ATT CAGA |
| *Eco*R I_AGT E42 | GAC TGC GTA CCA ATT CAGT |
| *Eco*R I_AGG E44 | GAC TGC GTA CCA ATT CAGG |
| *Hpa*II/*Msp*I Selective amplification primers |  |
| *Hpa* Ⅱ/ *Msp* Ⅰ-HMTGC | CAT GAG TCC TGC TCG GTGC |
| *Hpa* Ⅱ/ *Msp* Ⅰ-HMTCA | CAT GAG TCC TGC TCG GTCA |
| *Hpa* Ⅱ/ *Msp* Ⅰ-HMTCCA | CAT GAG TCC TGC TCG GTCCA |
| *Hpa* Ⅱ/ *Msp* Ⅰ-HMTCGA | CAT GAG TCC TGC TCG GTCGA |

**Table S4** The monthly mean data on weather parameters across 16 populations of *C. yanhusuo.*

| Populations | PRS_Avg  (hpa) | TEM_Avg  (℃) | TEM_Max  (℃) | TEM_Min  (℃) | RHU_Avg  (%) | PRE_Time  (mm) | SSH  (hour) |
| --- | --- | --- | --- | --- | --- | --- | --- |
| Cultivated |  |  |  |  |  |  |  |
| GZ | 984.2625 | 11.7125 | 27.7875 | -0.2875 | 79.6250 | 115.1750 | 136.0875 |
| QX | 1009.7125 | 13.7625 | 28.3000 | 3.2625 | 69.0000 | 118.3625 | 124.2000 |
| HZ | 960.4625 | 11.1000 | 22.8125 | 3.1125 | 73.3750 | 36.3625 | 102.2125 |
| CG | 963.0500 | 10.4125 | 23.3875 | 1.0875 | 78.2500 | 31.8500 | 127.1125 |
| HQ | 1019.8625 | 12.3750 | 25.5500 | 1.4000 | 74.8750 | 60.4375 | 139.9000 |
| JS | 1020.5750 | 11.4000 | 25.1875 | 0.4375 | 74.3750 | 73.8750 | 164.1250 |
| Natural |  |  |  |  |  |  |  |
| DP | 984.2625 | 11.7125 | 27.7875 | -0.2875 | 79.6250 | 115.1750 | 136.0875 |
| CH | 1006.4750 | 11.7875 | 27.6625 | -0.1000 | 75.0000 | 99.6000 | 142.7000 |
| DS | 1017.9500 | 11.7375 | 25.1250 | 0.0000 | 73.3750 | 58.8250 | 140.7750 |
| ZJ | 1016.9375 | 12.0250 | 25.2750 | 0.6750 | 68.2500 | 48.0375 | 168.8000 |
| HS | 1020.7125 | 12.3750 | 25.8750 | 1.1000 | 69.0000 | 90.4250 | 169.2375 |
| MS | 1016.7750 | 11.5500 | 25.1125 | 0.0125 | 70.5000 | 57.3500 | 173.6625 |
| DL | 1019.0500 | 11.2500 | 25.4375 | -2.2375 | 75.8750 | 98.8125 | 163.3625 |
| XY | 1007.0625 | 11.7500 | 26.4625 | 1.1500 | 66.1250 | 49.0500 | 127.5250 |
| SC | 1009.4375 | 11.6500 | 26.5000 | 0.0250 | 65.1250 | 60.1250 | 138.7375 |
| LY | 1017.1250 | 10.9625 | 24.5875 | -1.4375 | 74.0000 | 56.0625 | 155.0375 |

Note: TEM_Avg, average daily temperatures; TEM_Min, lowest daily temperatures; TEM_Max, highest daily temperatures, RHU_Avg, average daily relative humidity; PRS_Avg, average air pressure; SSH, total daily sunshine time; PRE_Time, total daily precipitation. The population DP and GZ shared one observation station.

**Table S5** Pairwise F_ST_ across 17 populations of *C. yanhusuo* using AFLP data

| Cultivated populations | | | | | | | Natural populations | | | | | | | | | |
| --- | --- | --- | --- | --- | --- | --- | --- | --- | --- | --- | --- | --- | --- | --- | --- | --- |
|  | CG | GZ | HQ | HZ | JS | QX | CH | DL | DP | DS | HS | XY | SC | LY | MS | TT |
| Cultivated populations | | | |  |  |  |  |  |  |  |  |  |  |  |  |  |
| GZ | 0.054 |  |  |  |  |  |  |  |  |  |  |  |  |  |  |  |
| HQ | 0.040 | 0.012 |  |  |  |  |  |  |  |  |  |  |  |  |  |  |
| HZ | 0.226 | 0.246 | 0.265 |  |  |  |  |  |  |  |  |  |  |  |  |  |
| JS | 0.061 | 0.060 | 0.100 | 0.160 |  |  |  |  |  |  |  |  |  |  |  |  |
| QX | 0.053 | 0.000 | 0.032 | 0.223 | 0.048 |  |  |  |  |  |  |  |  |  |  |  |
| Natural populations | | |  |  |  |  |  |  |  |  |  |  |  |  |  |  |
| CH | 0.527 | 0.525 | 0.518 | 0.420 | 0.457 | 0.505 |  |  |  |  |  |  |  |  |  |  |
| DL | 0.486 | 0.505 | 0.494 | 0.418 | 0.464 | 0.489 | 0.452 |  |  |  |  |  |  |  |  |  |
| DP | 0.518 | 0.518 | 0.515 | 0.406 | 0.461 | 0.502 | 0.256 | 0.423 |  |  |  |  |  |  |  |  |
| DS | 0.511 | 0.531 | 0.514 | 0.450 | 0.498 | 0.520 | 0.480 | 0.173 | 0.453 |  |  |  |  |  |  |  |
| HS | 0.482 | 0.488 | 0.475 | 0.451 | 0.460 | 0.480 | 0.501 | 0.409 | 0.469 | 0.393 |  |  |  |  |  |  |
| XY | 0.401 | 0.422 | 0.408 | 0.356 | 0.380 | 0.410 | 0.416 | 0.352 | 0.389 | 0.384 | 0.287 |  |  |  |  |  |
| SC | 0.496 | 0.510 | 0.500 | 0.451 | 0.484 | 0.503 | 0.520 | 0.299 | 0.490 | 0.253 | 0.352 | 0.368 |  |  |  |  |
| LY | 0.502 | 0.528 | 0.513 | 0.455 | 0.495 | 0.512 | 0.514 | 0.074 | 0.491 | 0.191 | 0.447 | 0.400 | 0.305 |  |  |  |
| MS | 0.423 | 0.428 | 0.423 | 0.310 | 0.363 | 0.409 | 0.244 | 0.341 | 0.271 | 0.376 | 0.368 | 0.290 | 0.413 | 0.412 |  |  |
| TT | 0.549 | 0.572 | 0.558 | 0.511 | 0.544 | 0.561 | 0.587 | 0.245 | 0.542 | 0.271 | 0.476 | 0.446 | 0.285 | 0.235 | 0.468 |  |
| ZJ | 0.345 | 0.355 | 0.349 | 0.297 | 0.285 | 0.340 | 0.421 | 0.398 | 0.436 | 0.429 | 0.440 | 0.365 | 0.435 | 0.434 | 0.349 | 0.476 |

**Table S6** Pairwise F_ST_ across 17 populations of *C. yanhusuo* using MSAP data.

| Cultivated populations | | | | | | | Natural populations | | | | | | | | | |
| --- | --- | --- | --- | --- | --- | --- | --- | --- | --- | --- | --- | --- | --- | --- | --- | --- |
|  | CG | GZ | HQ | HZ | JS | QX | CH | DL | DP | DS | HS | XY | SC | LY | MS | TT |
| Cultivated populations | | | |  |  |  |  |  |  |  |  |  |  |  |  |  |
| GZ | 0.203 |  |  |  |  |  |  |  |  |  |  |  |  |  |  |  |
| HQ | 0.196 | 0.029 |  |  |  |  |  |  |  |  |  |  |  |  |  |  |
| HZ | 0.131 | 0.281 | 0.245 |  |  |  |  |  |  |  |  |  |  |  |  |  |
| JS | 0.067 | 0.191 | 0.183 | 0.134 |  |  |  |  |  |  |  |  |  |  |  |  |
| QX | 0.186 | 0.000 | 0.030 | 0.263 | 0.183 |  |  |  |  |  |  |  |  |  |  |  |
| Natural populations | | |  |  |  |  |  |  |  |  |  |  |  |  |  |  |
| CH | 0.364 | 0.391 | 0.347 | 0.305 | 0.336 | 0.376 |  |  |  |  |  |  |  |  |  |  |
| DL | 0.407 | 0.419 | 0.388 | 0.374 | 0.382 | 0.404 | 0.308 |  |  |  |  |  |  |  |  |  |
| DP | 0.369 | 0.393 | 0.354 | 0.316 | 0.342 | 0.378 | 0.110 | 0.310 |  |  |  |  |  |  |  |  |
| DS | 0.417 | 0.420 | 0.388 | 0.388 | 0.391 | 0.405 | 0.333 | 0.182 | 0.331 |  |  |  |  |  |  |  |
| HS | 0.423 | 0.441 | 0.415 | 0.402 | 0.400 | 0.428 | 0.346 | 0.342 | 0.337 | 0.361 |  |  |  |  |  |  |
| XY | 0.407 | 0.425 | 0.389 | 0.369 | 0.381 | 0.411 | 0.318 | 0.278 | 0.319 | 0.321 | 0.236 |  |  |  |  |  |
| SC | 0.360 | 0.385 | 0.353 | 0.324 | 0.337 | 0.369 | 0.263 | 0.227 | 0.279 | 0.237 | 0.315 | 0.248 |  |  |  |  |
| LY | 0.367 | 0.382 | 0.348 | 0.336 | 0.345 | 0.366 | 0.283 | 0.133 | 0.275 | 0.192 | 0.264 | 0.265 | 0.236 |  |  |  |
| MS | 0.402 | 0.411 | 0.385 | 0.378 | 0.380 | 0.399 | 0.333 | 0.246 | 0.322 | 0.252 | 0.273 | 0.309 | 0.279 | 0.183 |  |  |
| TT | 0.413 | 0.425 | 0.395 | 0.380 | 0.386 | 0.411 | 0.325 | 0.149 | 0.327 | 0.149 | 0.359 | 0.303 | 0.234 | 0.161 | 0.265 |  |
| ZJ | 0.400 | 0.428 | 0.397 | 0.364 | 0.382 | 0.414 | 0.309 | 0.363 | 0.319 | 0.391 | 0.277 | 0.270 | 0.320 | 0.329 | 0.362 | 0.377 |

| Cultivated populations | | | | | | | Natural populations | | | | | | | | | |
| --- | --- | --- | --- | --- | --- | --- | --- | --- | --- | --- | --- | --- | --- | --- | --- | --- |
|  | CG | GZ | HQ | HZ | JS | QX | CH | DL | DP | DS | HS | XY | SC | LY | MS | TT |
| Cultivated populations | | | |  |  |  |  |  |  |  |  |  |  |  |  |  |
| GZ | 0.022 |  |  |  |  |  |  |  |  |  |  |  |  |  |  |  |
| HQ | 0.015 | 0.015 |  |  |  |  |  |  |  |  |  |  |  |  |  |  |
| HZ | 0.073 | 0.080 | 0.072 |  |  |  |  |  |  |  |  |  |  |  |  |  |
| JS | 0.025 | 0.025 | 0.026 | 0.046 |  |  |  |  |  |  |  |  |  |  |  |  |
| QX | 0.021 | 0.007 | 0.015 | 0.070 | 0.021 |  |  |  |  |  |  |  |  |  |  |  |
| Natural populations | | |  |  |  |  |  |  |  |  |  |  |  |  |  |  |
| CH | 0.174 | 0.187 | 0.151 | 0.099 | 0.122 | 0.169 |  |  |  |  |  |  |  |  |  |  |
| DL | 0.230 | 0.251 | 0.218 | 0.175 | 0.196 | 0.229 | 0.154 |  |  |  |  |  |  |  |  |  |
| DP | 0.197 | 0.208 | 0.175 | 0.115 | 0.145 | 0.189 | 0.048 | 0.153 |  |  |  |  |  |  |  |  |
| DS | 0.276 | 0.297 | 0.258 | 0.212 | 0.241 | 0.281 | 0.182 | 0.088 | 0.189 |  |  |  |  |  |  |  |
| HS | 0.214 | 0.220 | 0.196 | 0.201 | 0.191 | 0.212 | 0.194 | 0.205 | 0.200 | 0.193 |  |  |  |  |  |  |
| XY | 0.150 | 0.165 | 0.139 | 0.122 | 0.126 | 0.154 | 0.115 | 0.156 | 0.121 | 0.192 | 0.117 |  |  |  |  |  |
| SC | 0.261 | 0.275 | 0.249 | 0.219 | 0.237 | 0.265 | 0.230 | 0.159 | 0.237 | 0.138 | 0.169 | 0.190 |  |  |  |  |
| LY | 0.250 | 0.276 | 0.243 | 0.211 | 0.231 | 0.255 | 0.203 | 0.040 | 0.209 | 0.103 | 0.235 | 0.190 | 0.168 |  |  |  |
| MS | 0.160 | 0.175 | 0.141 | 0.091 | 0.114 | 0.157 | 0.047 | 0.134 | 0.068 | 0.167 | 0.163 | 0.096 | 0.206 | 0.185 |  |  |
| TT | 0.326 | 0.350 | 0.322 | 0.302 | 0.317 | 0.334 | 0.321 | 0.134 | 0.306 | 0.155 | 0.281 | 0.264 | 0.163 | 0.132 | 0.286 |  |
| ZJ | 0.113 | 0.117 | 0.103 | 0.093 | 0.081 | 0.104 | 0.122 | 0.175 | 0.152 | 0.208 | 0.203 | 0.137 | 0.217 | 0.205 | 0.124 | 0.269 |

**Table S7** Nei's distance across 17 populations of *C. yanhusuo* using AFLP data

**Table S8** Nei's distance across 17 populations of *C. yanhusuo* using MSAP data.

| Cultivated populations | | | | | | | Natural populations | | | | | | | | | |
| --- | --- | --- | --- | --- | --- | --- | --- | --- | --- | --- | --- | --- | --- | --- | --- | --- |
|  | CG | GZ | HQ | HZ | JS | QX | CH | DL | DP | DS | HS | XY | SC | LY | MS | TT |
| Cultivated populations | | | |  |  |  |  |  |  |  |  |  |  |  |  |  |
| GZ | 0.357 |  |  |  |  |  |  |  |  |  |  |  |  |  |  |  |
| HQ | 0.356 | 0.216 |  |  |  |  |  |  |  |  |  |  |  |  |  |  |
| HZ | 0.244 | 0.439 | 0.449 |  |  |  |  |  |  |  |  |  |  |  |  |  |
| JS | 0.225 | 0.349 | 0.334 | 0.306 |  |  |  |  |  |  |  |  |  |  |  |  |
| QX | 0.348 | 0.166 | 0.207 | 0.428 | 0.334 |  |  |  |  |  |  |  |  |  |  |  |
| Natural populations | | |  |  |  |  |  |  |  |  |  |  |  |  |  |  |
| CH | 0.612 | 0.653 | 0.632 | 0.626 | 0.624 | 0.595 |  |  |  |  |  |  |  |  |  |  |
| DL | 0.641 | 0.693 | 0.654 | 0.610 | 0.629 | 0.647 | 0.554 |  |  |  |  |  |  |  |  |  |
| DP | 0.579 | 0.616 | 0.604 | 0.598 | 0.619 | 0.615 | 0.268 | 0.529 |  |  |  |  |  |  |  |  |
| DS | 0.644 | 0.722 | 0.677 | 0.673 | 0.622 | 0.674 | 0.539 | 0.315 | 0.510 |  |  |  |  |  |  |  |
| HS | 0.612 | 0.647 | 0.630 | 0.622 | 0.615 | 0.623 | 0.539 | 0.529 | 0.494 | 0.523 |  |  |  |  |  |  |
| XY | 0.587 | 0.664 | 0.618 | 0.595 | 0.616 | 0.662 | 0.560 | 0.449 | 0.525 | 0.530 | 0.365 |  |  |  |  |  |
| SC | 0.595 | 0.634 | 0.617 | 0.574 | 0.572 | 0.629 | 0.582 | 0.421 | 0.566 | 0.362 | 0.532 | 0.457 |  |  |  |  |
| LY | 0.614 | 0.639 | 0.617 | 0.611 | 0.573 | 0.591 | 0.550 | 0.257 | 0.533 | 0.360 | 0.455 | 0.467 | 0.426 |  |  |  |
| MS | 0.600 | 0.622 | 0.595 | 0.606 | 0.577 | 0.604 | 0.627 | 0.432 | 0.613 | 0.377 | 0.452 | 0.527 | 0.417 | 0.383 |  |  |
| TT | 0.683 | 0.677 | 0.676 | 0.650 | 0.645 | 0.659 | 0.565 | 0.262 | 0.517 | 0.269 | 0.548 | 0.500 | 0.415 | 0.282 | 0.432 |  |
| ZJ | 0.598 | 0.622 | 0.609 | 0.581 | 0.613 | 0.618 | 0.557 | 0.544 | 0.507 | 0.598 | 0.351 | 0.405 | 0.530 | 0.571 | 0.564 | 0.564 |

**Table S9** Results of dbRDA correlating climatic variation to genetic, epigenetic, and alkaloid (cultivated populations only) variation.

| Environmental factors |  | Natural populations | | | | |  | Cultivated populations | | | | | | | |
| --- | --- | --- | --- | --- | --- | --- | --- | --- | --- | --- | --- | --- | --- | --- | --- |
|  |  | AFLP | |  | MSAP | |  | AFLP | |  | MSAP | |  | alkaloid | |
|  |  | r^2^ | *P* |  | r^2^ | *P* |  | r^2^ | *P* |  | r^2^ | *P* |  | r^2^ | *P* |
| PRS_Avg |  | 0.2887 | ^***^ |  | 0.4885 | ^***^ |  | 0.2012 | ^***^ |  | 0.2435 | ^***^ |  | 0.1107 | NS |
| TEM_Avg |  | 0.6857 | ^***^ |  | 0.4209 | ^***^ |  | 0.1024 | ^**^ |  | 0.4406 | ^***^ |  | 0.4869 | ^***^ |
| TEM_Max |  | 0.3148 | ^***^ |  | 0.5111 | ^***^ |  | 0.2476 | ^***^ |  | 0.6567 | ^***^ |  | 0.4952 | ^***^ |
| TEM_Min |  | 0.7478 | ^***^ |  | 0.4060 | ^***^ |  | 0.2212 | ^***^ |  | 0.1709 | ^***^ |  | 0.3852 | ^***^ |
| RHU_Avg |  | 0.5918 | ^***^ |  | 0.3554 | ^***^ |  | 0.0328 | NS |  | 0.0247 | NS |  | 0.5804 | ^***^ |
| PRE_Time |  | 0.1479 | ^***^ |  | 0.0701 | ^**^ |  | 0.1457 | ^***^ |  | 0.5108 | ^***^ |  | 0.4908 | ^***^ |
| SSH |  | 0.0020 | NS |  | 0.4639 | ^***^ |  | 0.4703 | ^***^ |  | 0.3170 | ^***^ |  | 0.0117 | NS |

Note: See Figure 6 for abbreviations of environmental factors; r^2^ represents the determination coefficient of the climatic explanatory and response variables.t; *P* values are indicated as: ^*^ *P* < 0.05; ^**^ *P* < 0.01; ^***^ *P* < 0.001, NS is not significant.
